# Preventing resistance development in infections by OXA β-lactamase-producing *Pseudomonas aeruginosa*: correlating clinical outcomes with hollow-fibre model input

**DOI:** 10.1093/jac/dkaf464

**Published:** 2025-12-16

**Authors:** María M Montero, Almudena Fernández-Muñoz, Sandra Domene-Ochoa, Silvia Castañeda, Inmaculada López-Montesinos, Sara Cortes-Lara, Sonia Luque, Maria Asunción Colomar, Xavier Mulet, Carla López-Causapé, Luisa Sorlí, Eduardo Padilla, Juan P Horcajada, Antonio Oliver

**Affiliations:** Infectious Diseases Service, Hospital del Mar. Infectious Pathology and Antimicrobials Research Group (IPAR), Hospital del Mar Research Institute (IMIM), Barcelona, Spain; Department of Medicine and Life Sciences (MELIS), Universitat Pompeu Fabra, Barcelona, Spain; CIBER of Infectious Diseases (CIBERINFEC CB21/13/00002 and CB21/13/00099), Institute of Health Carlos III, Madrid, Spain; Servicio de Microbiología y Unidad de Investigación, Hospital Son Espases, IdISBa, Palma de Mallorca, Spain; Infectious Diseases Service, Hospital del Mar. Infectious Pathology and Antimicrobials Research Group (IPAR), Hospital del Mar Research Institute (IMIM), Barcelona, Spain; CIBER of Infectious Diseases (CIBERINFEC CB21/13/00002 and CB21/13/00099), Institute of Health Carlos III, Madrid, Spain; Infectious Diseases Service, Hospital del Mar. Infectious Pathology and Antimicrobials Research Group (IPAR), Hospital del Mar Research Institute (IMIM), Barcelona, Spain; Infectious Diseases Service, Hospital del Mar. Infectious Pathology and Antimicrobials Research Group (IPAR), Hospital del Mar Research Institute (IMIM), Barcelona, Spain; CIBER of Infectious Diseases (CIBERINFEC CB21/13/00002 and CB21/13/00099), Institute of Health Carlos III, Madrid, Spain; Servicio de Microbiología y Unidad de Investigación, Hospital Son Espases, IdISBa, Palma de Mallorca, Spain; CIBER of Infectious Diseases (CIBERINFEC CB21/13/00002 and CB21/13/00099), Institute of Health Carlos III, Madrid, Spain; Pharmacy Service, Hospital del Mar, Barcelona, Spain; CIBER of Infectious Diseases (CIBERINFEC CB21/13/00002 and CB21/13/00099), Institute of Health Carlos III, Madrid, Spain; Medicina Intensiva, Hospital Son Espases, IdISBa, Palma de Mallorca, Spain; CIBER of Infectious Diseases (CIBERINFEC CB21/13/00002 and CB21/13/00099), Institute of Health Carlos III, Madrid, Spain; Servicio de Microbiología y Unidad de Investigación, Hospital Son Espases, IdISBa, Palma de Mallorca, Spain; CIBER of Infectious Diseases (CIBERINFEC CB21/13/00002 and CB21/13/00099), Institute of Health Carlos III, Madrid, Spain; Servicio de Microbiología y Unidad de Investigación, Hospital Son Espases, IdISBa, Palma de Mallorca, Spain; Infectious Diseases Service, Hospital del Mar. Infectious Pathology and Antimicrobials Research Group (IPAR), Hospital del Mar Research Institute (IMIM), Barcelona, Spain; Department of Medicine and Life Sciences (MELIS), Universitat Pompeu Fabra, Barcelona, Spain; CIBER of Infectious Diseases (CIBERINFEC CB21/13/00002 and CB21/13/00099), Institute of Health Carlos III, Madrid, Spain; Department of Medicine and Life Sciences (MELIS), Universitat Pompeu Fabra, Barcelona, Spain; Microbiology Service, Laboratori de Referència de Catalunya, Barcelona, Spain; Infectious Diseases Service, Hospital del Mar. Infectious Pathology and Antimicrobials Research Group (IPAR), Hospital del Mar Research Institute (IMIM), Barcelona, Spain; Department of Medicine and Life Sciences (MELIS), Universitat Pompeu Fabra, Barcelona, Spain; CIBER of Infectious Diseases (CIBERINFEC CB21/13/00002 and CB21/13/00099), Institute of Health Carlos III, Madrid, Spain; CIBER of Infectious Diseases (CIBERINFEC CB21/13/00002 and CB21/13/00099), Institute of Health Carlos III, Madrid, Spain; Servicio de Microbiología y Unidad de Investigación, Hospital Son Espases, IdISBa, Palma de Mallorca, Spain

## Abstract

**Background:**

Infections caused by XDR *Pseudomonas aeruginosa* significantly limit treatment options. Although ceftolozane/tazobactam and ceftazidime/avibactam have emerged as promising alternatives, increasing resistance has been reported. This study describes three critically ill patients with ventilator-associated pneumonia caused by OXA-producing XDR *P. aeruginosa* that developed resistance to both agents during therapy.

**Methods:**

Antibiotic exposure and resistance were monitored clinically and in a hollow-fibre infection model (HFIM) to evaluate different ceftazidime/avibactam and ceftolozane/tazobactam regimens. Drug concentrations, bacterial burden and resistant mutants were assessed. Whole-genome sequencing and resistance profiling were performed on both clinical and HFIM-derived isolates.

**Results:**

Initial patient isolates were susceptible to both antibiotics. One belonged to ST179 (OXA-10 producer), while two were ST235 (OXA-2 or OXA-2 + OXA-10 producers). Resistance emerged during therapy in all cases. In the HFIM, continuous infusion of ceftolozane/tazobactam plus meropenem achieved bacterial eradication for ST179 within 8 h. For ST235 isolates, high-exposure ceftazidime/avibactam regimens (4-h or continuous infusion) achieved bacterial eradication and prevented regrowth. In contrast, low-exposure 2-h infusions allowed bacterial rebound and resistance selection. Mechanisms of resistance were similar across clinical and HFIM isolates, involving overexpression or structural modification of OXA enzymes, except in one ST235 HFIM derived-isolate, in which resistance development was caused by an AmpC Ω-loop mutation. Pharmacokinetic validation confirmed accurate drug exposure in the model.

**Conclusions:**

These findings underscore the importance of optimized dosing strategies, particularly high-concentration and prolonged infusions, in eradicating and preventing resistance development in OXA-producing *P. aeruginosa* infections.

## Introduction

*Pseudomonas aeruginosa* is among the main causes of hospital-acquired and chronic infections and is associated with high antimicrobial resistance, morbidity and mortality.^1^ Antibiotic-resistant *P. aeruginosa* infections are estimated to be associated with over 300 000 annual deaths.^2^ This growing threat results from its capacity to develop resistance through chromosomal mutations and the increasing prevalence of transferable resistance determinants.^3,4^ These mechanisms lead to complex resistance profiles as defined by the ECDC (MDR, XDR and PDR) or by the infectious Diseases Society of America (IDSA) [difficult to treat resistance (DTR)].^5,6^ XDR/DTR global lineages, denominated high-risk clones, are disseminated in hospitals worldwide.^7,8^

Ceftolozane/tazobactam and ceftazidime/avibactam are preferred treatment options for XDR *P. aeruginosa* infections. However, their clinical efficacy is increasingly threatened by the emergence of resistance during treatment.^9^ The most relevant chromosomal target for mutation-driven resistance is the catalytic centre (the Ω-loop) of the intrinsic β-lactamase AmpC.^10,11^ However, there is growing evidence of the relevance of horizontally acquired OXA β-lactamases.^12^

OXA-2 and OXA-10 are among the most frequent transferable β-lactamases in *P. aeruginosa*.^13^ Although several ESBL variants of these enzymes have been described over the last decades, their clinical relevance has often been overlooked. The introduction of ceftolozane/tazobactam and ceftazidime/avibactam appears to be changing this, as selection of ESBL variants has been reported as a frequent cause of resistance during treatment in OXA-2 or OXA-10 producing *P. aeruginosa* strains.^10,14^

Of the four resistance cases documented in a recent clinical study (Pseudonova cohort, manuscript in preparation), one was driven by AmpC Ω-loop mutations, while the other three were related to OXA enzymes, either by overexpression or by mutations in the catalytic centre leading to the emergence of extended spectrum ESBL variants.

The hollow-fibre infection model (HFIM) provides a robust platform to simulate pharmacokinetic and pharmacodynamic (PK/PD) antibiotic conditions, enabling evaluation of bacterial responses under varying antibiotic exposures, resistance development and dosing strategies. A previous HFIM study documented the case of AmpC Ω-loop mutation, leading to treatment optimization and preventing resistance.^15^

This study investigates three clinical cases of OXA-2 and/or OXA-10 XDR *P. aeruginosa* isolates from the Pseudonova cohort that developed resistance during treatment. Using HFIM, we analysed bacterial dynamics, resistance mechanisms and pharmacokinetics under different ceftolozane/tazobactam and ceftazidime/avibactam dosing regimens. These findings aim to support therapeutic optimization and resistance prevention in XDR *P. aeruginosa* infections.

## Material and methods

### Bacterial isolates

The clinical *P. aeruginosa* isolates were obtained from a prospective multicentre observational study conducted at third-level reference centres in Spain, known as PseudoNOVA, which took place between May 2018 and 2020 (Pseudonova cohort, manuscript in preparation), and included adult patients with XDR *P. aeruginosa* infections who received ≥48 h of targeted therapy and had available pre- and post-treatment isolates. Its main objectives were to compare clinical outcomes, toxicity, microbiological eradication and resistance emergence in patients treated with ceftolozane/tazobactam or ceftazidime/avibactam versus colistin, and to correlate these results with plasma antibiotic concentrations. As part of that study, the complete cohort of isolates were characterized at phenotypic (broth microdilution susceptibility testing) and genomic (WGS) level. We used the three isolates producing OXA-2 and/or OXA-10 recovered from three patients that developed resistance upon treatment with ceftazidime/avibactam or ceftolozane/tazobactam. The strains were stored frozen in 20% glycerol at −80°C.

### Antimicrobial agents

Clinical vials were used to prepare the antibiotics, with each powder vial reconstituted with injectable water solution, in order to reproduce *in vitro* the exact composition, excipients and stability conditions of the drugs used in clinical practice. The antibiotics used were: ceftolozane/tazobactam (Zerbaxa^®^, provided by Merck & Co., Inc., Kenilworth, NJ, USA) 1 g/0.5 g powder for infusion; meropenem (Meropenem SUN^®^, provided by Sun Pharmaceutical Industries Europe B.V., Hoofddorp, Netherlands) 1 g powder for infusion; ceftazidime/avibactam (Zavicefta^®^, provided by Pfizer, Ringaskiddy, County Cork, Ireland) 2 g/0.5 g powder for infusion; aztreonam (Azactam^®^, provided by Bristol-Myers Squibb S.A., Madrid, Spain) 1 g powder for infusion. The dosing regimens simulated in the HFIM, along with their corresponding pharmacokinetic parameters,^16–18^ are summarized in Table 1.

**Table 1.** Dosing regimens simulated in the hollow-fibre infection model by isolate, with corresponding pharmacokinetic parameters

Isolate (ST)	Antibiotic	Dose/infusion	^a^ *C*_max_/*C*_ss_ (mg/L)	AUC (µg·h/mL)	t½ (h)	Notes
12-008 (ST179)	C/T	2/1 g q8h (4-h inf)	54	857	3/1	Clinical dose
	C/T	2/1 g CI	54	1300	3/1	Same *C*_ss_ as clinical dose
	Meropenem	2 g q8h (4-h inf)	33	424	1	—
	C/T + Meropenem	CI + 2 g q8h (4-h inf)	—	—	—	Combination
12-016 (ST235)	CZA	2/0.5 g q8h (2-h inf)	16	141	2	2× MIC
	CZA	2/0.5 g q8h (4-h inf)	112	1804	2	Clinical dose
	CZA	2/0.5 g CI	16	311	2	2× MIC
	CZA	2/0.5 g CI	112	1566	2	Same *C*_ss_ as clinical dose
12-017 (ST235)	CZA	2/0.5 g q8h (2-h inf)	12	141	2	Clinical dose
	CZA	2/0.5 g q8h (4-h inf)	64	898	2	8× MIC
	CZA	2/0.5 g CI	12	311	2	Same *C*_ss_ as clinical dose
	CZA	2/0.5 g CI	40	981	2	5× MIC
	CZA	2/0.5 g CI	64	1558	2	8× MIC

AUC, area under the curve; C/T, ceftolozane/tazobactam; CI, continuous infusion; *C*_ss_, steady-state concentration; CZA, ceftazidime/avibactam; inf, infusion; MIC, minimum inhibitory concentration; q8h, every 8 h; ST, Sequence type; t½, half-life.

^a^ *C*_max_ (intermittent) and *C*_ss_ (continuous) were intentionally matched to allow direct comparison of equivalent peak exposures between regimens. The continuous infusion regimens correspond to total daily doses of 6/3 g over 24 h, whereas the intermittent regimens simulate 2/1 g every 8 h. Simulations were based on plasma concentrations measured in critically ill patients.

### HFIM

Duplicate HFIM assays were conducted to compare different conditions, lasting between 8 and 14 days depending on the regimen followed by the patient, with a control without antibiotics in each case. The HFIM was performed as previously described,^16^ with experimental details provided below. Drug-free control runs were performed using the original parental clinical isolate, without exposure to antimicrobials, to assess baseline growth. Polyethersulfone hemofilters were used for the HFIM cartridges, with a volume of 50 mL (Aquamax HF03, Nikkiso, Belgium).^19^ The experiment was conducted in a humidified incubator at 37°C. Antibiotics were directly administered into the central reservoir with a separate infusion pump to achieve the desired concentrations. Antibiotic-free growth medium (cation-adjusted Mueller-Hinton broth, CAMHB) was continuously infused into the central compartment to simulate drug clearance in humans, and an equal volume was extracted from the same compartment to maintain an isovolumetric system. The bacterial suspension was inoculated in the extracellular compartment of the cartridges, where it was exposed to different antibiotic concentrations. Samples were taken from the cartridges at the following time points: 0, 8, 24, 48, 72, 96, 144, 168, 192 (for the 12-017 ST235 isolate with an 8-day duration), 216, 240, 312 and 336 h. Aliquots were centrifuged for 3 min at 13 000 rpm, supernatants were discarded and pellets were washed and resuspended in saline solution. Serial dilutions were performed diluted in CAMHB, plated on TSA plates and incubated overnight at 37°C for subsequent colony-forming unit (cfu/mL) counts. Results were converted to a base-10 logarithmic scale.

### Pharmacokinetic studies

Clinical pharmacokinetic concentrations were obtained from patients included in the Pseudonova study, in whom plasma levels of ceftolozane/tazobactam and ceftazidime/avibactam were measured as part of therapeutic drug monitoring. The measured values, which occasionally differed from concentrations reported in healthy volunteers, reflected the variability observed in critically ill patients and were used to define the clinical exposure profiles simulated in the HFIM. This variability was intentionally reproduced to capture real-world pharmacokinetic conditions. The continuous infusion regimen (6/3 g over 24 h) reproduced the total daily dose administered to Pseudonova patients, whereas the intermittent regimen simulated 2/1 g every 8 h. For comparative purposes, the intermittent and continuous regimens were designed so that the maximum concentration (*C*_max_) of the intermittent schedule corresponded to the steady-state concentration (*C*_ss_) achieved in continuous infusion (e.g. both 54 mg/L). This approach allowed evaluation of equivalent peak exposures under different dosing modes.

Throughout the experiment, antibiotic samples were taken from the peripheral compartment of the cartridge and stored at −80°C until analysis. Antibiotic concentrations in the HFIM were sampled at predefined time points within each dosing interval to enable direct experimental determination of the *C*_max_, the minimum concentration (*C*_min_) and *C*_ss_. In this study, ‘*C*_ss_’ refers to the plateau concentration achieved during continuous infusion once steady state was reached. For comparative purposes, the *C*_max_ of intermittent regimens was matched to this *C*_ss_ value to allow evaluation of equivalent peak exposures under different infusion modes. For intermittent regimens, samples were collected at pre-dose (0 h, trough/*C*_min_), at the end of infusion (EoI; 2 h or 4 h depending on regimen, corresponding to *C*_max_) and at intermediate post-infusion times (typically 1 h and 4–6 h after EoI) with the 8 h time point representing the next *C*_min_. For continuous infusion regimens, samples were taken at 0, 4, 8, 12 and 24 h (and subsequently at 24 h intervals) to confirm attainment and maintenance of steady state. Additional daily samples (24, 48, 72, 96, 144, 168 h and thereafter as required by experiment duration up to 336 h) were collected to assess reproducibility (Table S1, available as Supplementary data at *JAC* Online). Pharmacokinetic sampling and validation were performed in duplicate independent HFIM runs for each dosing regimen. In the Pseudonova study, plasma concentrations were measured at specific time points. HFIM simulations were calibrated to reproduce these measured concentrations. The correspondence between clinical and simulated concentrations is summarized in Table S2.

Samples were analysed by HPLC (Waters e2695 separations module, Milford, MA, USA) with an ultraviolet light detector (2489 UV/Vis detector, Milford, MA, USA) in CAMHB culture medium. The agreement between theoretical concentrations and observed concentrations was evaluated using the Bland–Altman graphical method, which assesses bias by comparing means and estimating 95% agreement intervals.^20^

### Monitoring of resistance emergence

To assess the emergence of resistance, aliquots of the bacterial suspension extracted from the extracapillary compartment of the HFIM cartridges were sampled at each pharmacodynamic time point (0, 8, 24, 48, 72, 96, 144, 168, 192, 216, 240, 312 and 336 h). Each sample was serially diluted and plated both on drug-free TSA plates (for total cfu enumeration) and on TSA plates supplemented with the corresponding antibiotic (ceftolozane/tazobactam or ceftazidime/avibactam) at 2×, 4×, 8× and 16× the initial MIC value of the starting isolate. The emergence of resistant colonies was recorded when growth was observed. Colonies from antibiotic-supplemented plates were subcultured and subjected to MIC testing using the MicroScan WalkAway 96 Plus system (Beckman Coulter, NMDRM1 panel) to determine MIC shifts relative to the parental strain. Mutant frequencies were calculated as the ratio of cfu growing on antibiotic-supplemented plates to total cfu on drug-free TSA at each time point.

### Characterization of resistant mutants

#### Susceptibility testing

MICs of piperacillin/tazobactam, ceftazidime, cefepime, ceftazidime/avibactam, ceftolozane/tazobactam, imipenem, meropenem, aztreonam, ciprofloxacin, tobramycin, amikacin and colistin were determined by broth microdilution using Sensititre^™^ custom panels (Plate Code: FRCNRP2, Thermo Fisher Diagnostics, S.LU) following EUCAST guidelines and 2024 clinical breakpoints (www.eucast.org). In addition, MICs of imipenem/relebactam (0.125–64 mg/L) were determined by in-house broth microdilution, while those of cefiderocol (0.03–16 mg/L) were determined using iron-depleted cation-adjusted Mueller–-Hinton broth, following EUCAST guidelines.

#### Whole genome sequencing

Previously defined and validated protocols were used with slight modifications (summarized in Supplementary Methods).^21^

#### Quantification of the expression of *bla*_OXA-10_

mRNA of *bla*_OXA-10_ gene was quantified through real-time RT-PCR according to previously described protocols (summarized in Supplementary Methods).^22,23^ To estimate the relative copy number, the average read coverage depths of *bla*_OXA-10_ gene along with that of the chromosomic housekeeping gene *rpsL* were obtained from the pileup files. The ratio of coverage between the resistance gene and *rpsL* was used to estimate the relative copy number of *bla*_OXA-10_ and results were normalized to the parental clinical strain.

#### Data availability

Genomic sequences obtained in this work have been deposited in the European Nucleotide Archive under project number PRJEB86485.

## Results

### *In vitro* susceptibility and resistance mechanisms developed in the tested clinical isolates

The initial susceptibility profile and molecular characterization of the isolates analysed from the PseudoNOVA study (Pseudonova cohort, manuscript in preparation) are detailed in Tables 2–4. The 12-008 (ST179) isolate obtained from bronchial aspirate (BAS) was susceptible (S) to ceftolozane/tazobactam (MIC 2 mg/L) and susceptible increased exposure (I) for meropenem (MIC 8 mg/L), and it carried an OXA-10 β-lactamase (Table 2). Ceftolozane/tazobactam- and meropenem-resistant mutants were recovered after treatment, and the underlying resistance mechanism was the hyperproduction of OXA-10 (Table 2). As shown in Table 2, increased expression was, at least partially, explained by increased copy numbers of *bla*_OXA-10_. The 12-016 (ST235) isolate, also obtained from BAS, was susceptible to ceftazidime/avibactam (MIC 4 mg/L) and I for aztreonam (MIC 16 mg/L) and carried both OXA-2 and OXA-10 β-lactamases (Table 3). Ceftazidime/avibactam-resistant mutants were recovered after treatment, and the underlying resistance mechanism was the acquisition of an extended-spectrum mutation (W159R) in OXA-2 leading to OXA-226 (Table 3). The 12-017 (ST235) isolate, obtained from sputum, was susceptible to ceftazidime/avibactam (MIC 8 mg/L) and carried an OXA-2 β-lactamase (Table 4). Ceftazidime/avibactam-resistant mutants were recovered after treatment, and the underlying resistance mechanism was the acquisition of an extended-spectrum mutation (N148K) in OXA-2 leading to OXA-838 (Table 3).

**Table 2.** Phenotypic and genomic characterization of patient 12-008 *P. aeruginosa* clinical isolates and derived resistant mutants

PA Isolates 12-008 (ST179)^a^	Treatment	MIC (mg/L)^b^										Resistome summary	*bla*_OXA-10_ expression ± SD	Relative number of reads for *bla*_OXA-10_^c^
		P/T4 (R > 16)	TAZ (R > 8)	C/T (S ≤ 4)		FEP (R > 8)	IMI (R > 4)	MER (R > 8)	AZT (R > 16)	FDC (R > 2)	IMR (R > 2)			
Parental	C/T	64	2	0.5-1	1	8	4	8	8	0.06	1	OXA-10, *aadA1*, *aac(6*′*)-Ib_Hangzhou*, *qnrVC1*, *dfrA14*, *oprD* (L355Q), *phoQ* (S300R), *parS* (V291L)	1	1
Clinical mutant 1	C/T	256	2	4	1.4	32	4	16	16	0.06	1	OXA-10, *aadA1*, *aac(6*′*)-Ib_Hangzhou*, *qnrVC1*, *dfrA14*, *oprD* (L355Q), *phoQ* (S300R), *parS* (V291L)	7.1 ± 0.1	1.4
Clinical mutant 2	C/T	>256	2	8	4.0	64	4	32	32	0.12	2	OXA-10, *aadA1*, *aac(6*′*)-Ib_Hangzhou*, *qnrVC1*, *dfrA14*, *oprD* (L355Q), *phoQ* (S300R), *parS* (V291L)	136.9 ± 5.6	4.0
HFIM_1_1	C/T 4 h infusion: 144h	256	2	4	0.8	16	8	16	16	0.06	0.5	OXA-10, *aadA1*, *aac(6*′*)-Ib_Hangzhou*, *qnrVC1*, *dfrA14*, *oprD* (L355Q), *phoQ* (S300R), *parS* (V291L)	8.3 ± 4.03	0.8
HFIM_1_2	C/T 4 h infusion: 168h	256	2	4	0.8	16	4	16	16	0.06	1	OXA-10, *aadA1*, *aac(6*′*)-Ib_Hangzhou*, *qnrVC1*, *dfrA14*, *oprD* (L355Q), *phoQ* (S300R), *parS* (V291L)	10.0 ± 3.1	0.8
HFIM_1_3	C/T 4 h infusion: 216h	256	2	4	1.2	16	8	16	16	0.06	1	OXA-10, *aadA1*, *aac(6*′*)-Ib_Hangzhou*, *qnrVC1*, *dfrA14*, *oprD* (L355Q), *phoQ* (S300R), *parS* (V291L)	11.2 ± 0.7	1.2
HFIM_2_1	C/T 4 h infusion: 168h	>256	2	8	3.1	64	8	32	32	0.12	2	OXA-10, *aadA1*, *aac(6*′*)-Ib_Hangzhou*, *qnrVC1*, *dfrA14*, *oprD* (L355Q), *phoQ* (S300R), *parS* (V291L)	53.7 ± 0.4	3.1
HFIM_2_2	C/T 4 h infusion: 216h	>256	2	8	2.8	32	8	32	32	0.12	2	OXA-10, *aadA1*, *aac(6*′*)-Ib_Hangzhou*, *qnrVC1*, *dfrA14*, *oprD* (L355Q), *phoQ* (S300R), *parS* (V291L)	61.7 ± 12.2	2.8
HFIM_2_3	MER 4 h infusion: 168h	256	2	8	2.1	32	8	32	32	0.12	2	OXA-10, *aadA1*, *aac(6*′*)-Ib_Hangzhou*, *qnrVC1*, *dfrA14*, *oprD* (L355Q), *phoQ* (S300R), *parS* (V291L)	62.8 ± 16.1	2.1
HFIM_2_4	MER 4 h infusion: 216h	>256	2	8	2.3	32	8	32	32	0.06	2	OXA-10, *aadA1*, *aac(6*′*)-Ib_Hangzhou*, *qnrVC1*, *dfrA14*, *oprD* (L355Q), *phoQ* (S300R), *parS* (V291L)	61.8 ± 14.1	2.3

^a^HFIM, hollow-fibre infection model mutants.

^b^P/T4, piperacillin-tazobactam; TAZ, ceftazidime; C/T, ceftolozane-tazobactam; CZA, ceftazidime-avibactam; FEP, cefepime; IMI, imipenem; MER, meropenem; AZT, aztreonam; FDC, cefiderocol; IMR, imipenem-relebactam. Ciprofloxacin, amikacin, tobramycin and colistin were also tested. All the strains resulted susceptible. All the strains resulted resistant for ciprofloxacin and tobramycin, and susceptible for amikacin and colistin.

^c^To estimate the relative copy number, the average read coverage depths of *bla*_OXA-10_ gene along with that of the chromosomic housekeeping gene *rpsL* were obtained from the pileup files. The ratio of coverage between the resistance gene and *rpsL* was used to estimate the relative copy number of *bla*OXA-10 and results were normalized to the parental clinical strain.

**Table 3.** Phenotypic and genomic characterization of patient 12-016 *P. aeruginosa* clinical isolates and derived resistant mutants

PA isolates 12-016 (ST235)^a^	Treatment	MIC (mg/L)^b^										Resistome summary^c^
		P/T4 (R > 16)	TAZ (R > 8)	C/T (S ≤ 4)	CZA (S ≤ 8)	FEP (R > 8)	IMI (R > 4)	MER (R > 8)	AZT (R > 16)	FDC (R > 2)	IMR (R > 2)	
Parental	CZA	128	8	4	4	16	16	64	16	0.25	4	OXA-2, OXA-10, *aac(6*′*)-Ib_Hangzhou*, *aacA7*, *aacA31*, *aadA1*, *aadA6*, *dfrA14*, *qnrVC1*, *mexR* (K44M), Δ*oprD*, *mexZ* (V48A), *gyrA* (T83I), *pmrB* (V344M), *parC* (S87L)
Clinical mutant	CZA	64	32	32	>32	16	16	16	16	0.12	4	**OXA-2 (W159R; OXA-226)**, OXA-10, *aac(6*′*)-Ib_Hangzhou*, *aacA7*, *aacA31*, *aadA1*, *aadA6*, *dfrA14*, *qnrVC1*, *mexR* (K44M), Δ*oprD*, *mexZ* (V48A), *gyrA* (T83I), *pmrB* (V344M), *parC* (S87L)
HFIM_1_1	CZA 2 h infusion: 144h	256	64	32	32	16	16	32	32	0.25	4	**OXA-2 (W159R; OXA-226)**, OXA-10, *aac(6*′*)-Ib_Hangzhou*, *aacA7*, *aacA31*, *aadA1*, *aadA6*, *dfrA14*, *qnrVC1*, *mexR* (K44M), Δ*oprD*, *mexZ* (V48A), *gyrA* (T83I), *pmrB* (V344M), *parC* (S87L)
HFIM_1_2	CZA 2 h infusion: 168h	256	64	>32	32	16	16	32	16	0.25	4	**OXA-2 (W159R; OXA-226)**, OXA-10, *aac(6*′*)-Ib_Hangzhou*, *aacA7*, *aacA31*, *aadA1*, *aadA6*, *dfrA14*, *qnrVC1*, *mexR* (K44M), Δ*oprD*, *mexZ* (V48A), *gyrA* (T83I), *pmrB* (V344M), *parC* (S87L)
HFIM_ 1_3	CZA 2 h infusion: 192h	256	32	32	32	16	16	32	32	0.25	4	**OXA-2 (W159R; OXA-226)**, OXA-10, *aac(6*′*)-Ib_Hangzhou*, *aacA7*, *aacA31*, *aadA1*, *aadA6*, *dfrA14*, *qnrVC1*, *mexR* (K44M), Δ*oprD*, *mexZ* (V48A), *gyrA* (T83I), *pmrB* (V344M), *parC* (S87L)
HFIM_2_1	CZA 2 h infusion: 120h	>256	>64	>32	>32	>64	32	32	64	1	4	OXA-2, **OXA-10 (G157D; OXA-14)**, *aac(6*′*)-Ib_Hangzhou*, *aacA7*, *aacA31*, *aadA1*, *aadA6*, *dfrA14*, *qnrVC1*, *mexR* (K44M), Δ*oprD*, *mexZ* (V48A), *gyrA* (T83I), *pmrB* (V344M), *parC* (S87L)
HFIM_2_2	CZA 2 h infusion: 144h	256	>64	>32	>32	64	16	32	64	1	4	OXA-2, **OXA-10 (G157D; OXA-14)**, *aac(6*′*)-Ib_Hangzhou*, *aacA7*, *aacA31*, *aadA1*, *aadA6*, *dfrA14*, *qnrVC1*, *mexR* (K44M), Δ*oprD*, *mexZ* (V48A), *gyrA* (T83I), *pmrB* (V344M), *parC* (S87L)
HFIM_2_3	CZA 2 h infusion: 168h	256	>64	32	>32	64	16	32	32	2	4	OXA-2, **OXA-10 (G157D; OXA-14)**, *aac(6*′*)-Ib_Hangzhou*, *aacA7*, *aacA31*, *aadA1*, *aadA6*, *dfrA14*, *qnrVC1*, *mexR* (K44M), Δ*oprD*, *mexZ* (V48A), *gyrA* (T83I), *pmrB* (V344M), *parC* (S87L)
HFIM_2_4	CZA IC: 120h	256	>64	32	>32	64	16	32	32	2	4	OXA-2, **OXA-10 (N143S, G157D; OXA-11)**, *aac(6*′*)-Ib_Hangzhou*, *aacA7*, *aacA31*, *aadA1*, *aadA6*, *dfrA14*, *qnrVC1*, *mexR* (K44M), Δ*oprD*, *mexZ* (V48A), *gyrA* (T83I), *pmrB* (V344M), *parC* (S87L)
HFIM_2_5	CZA IC: 144h	256	>64	32	>32	64	16	16	32	1	4	OXA-2, **OXA-10 (N143S, G157D; OXA-11)**, *aac(6*′*)-Ib_Hangzhou*, *aacA7*, *aacA31*, *aadA1*, *aadA6*, *dfrA14*, *qnrVC1*, *mexR* (K44M), Δ*oprD*, *mexZ* (V48A), *gyrA* (T83I), *pmrB* (V344M), *parC* (S87L)
HFIM_2_6	CZA IC: 168h	128	>64	>32	>32	64	16	32	64	1	4	OXA-2, **OXA-10 (N143S, G157D; OXA-11)**, *aac(6*′*)-Ib_Hangzhou*, *aacA7*, *aacA31*, *aadA1*, *aadA6*, *dfrA14*, *qnrVC1*, *mexR* (K44M), Δ*oprD*, *mexZ* (V48A), *gyrA* (T83I), *pmrB* (V344M), *parC* (S87L)

^a^HFIM, hollow-fibre infection model mutants.

^b^P/T4, piperacillin-tazobactam; TAZ, ceftazidime; C/T, ceftolozane-tazobactam; CZA, ceftazidime-avibactam; FEP, cefepime; IMI, imipenem; MER, meropenem; AZT, aztreonam; FDC, cefiderocol; IMR, imipenem-relebactam. Ciprofloxacin, amikacin, tobramycin and colistin were also tested. All the strains resulted resistant for ciprofloxacin, amikacin and tobramycin, and susceptible for colistin.

^c^Acquired mutations are highlighted in bold.

**Table 4.** Phenotypic and genomic characterization of patient 12-017 *P. aeruginosa* clinical isolates and derived resistant mutants

PA isolate 12-017 (ST235)^a^	Treatment			MIC (mg/L)^b^								Resistome summary^c^
		P/T4 (R > 16)	TAZ (R > 8)	C/T (S ≤ 4)	CZA (S ≤ 8)	FEP (R > 8)	IMI (R > 4)	MER (R > 8)	AZT (R > 16)	FDC (R > 2)	IMR (R > 2)	
Parental Strain	CZA	256	64	1	8	32	16–32	16–32	64	0.5	2	OXA-2, *aacA7*, *aacA31*, *aadA6*, *mexR* (K44M), *oprD* (nt209Δ1), *mexZ* (V48A), *dacB* (P70L), *gyrA* (T83I), *ftsI* (G216S), *pmrB* (V344M), *parC* (S87L)
Clinical mutant	CZA	256	64	8–16	32	32	16–32	32	64	0.5	2	**OXA-2 (N148K; OXA-838)**, *aacA7*, *aacA31*, *aadA6*, *mexR* (K44M), *oprD* (nt209Δ1), *mexZ* (V48A), *dacB* (P70L), *gyrA* (T83I), *ftsI* (G216S), *pmrB* (V344M), *parC* (S87L)
HFIM_1_1	CZA 2 h infusion: 120h	256	>64	>32	32	32	32	32	32	0.5	2	OXA-2, *aacA7*, *aacA31*, *aadA6*, *mexR* (K44M), *oprD* (nt209Δ1), *mexZ* (V48A), *dacB* (P70L), *gyrA* (T83I), ***ampC* (G183D)**, *ftsI* (G216S), *pmrB* (V344M), *parC* (S87L)
HFIM_1_2	CZA 2 h infusion: 144h	>256	64	32	32	32	32	64	32	0.5	2–4	OXA-2, *aacA7*, *aacA31*, *aadA6*, *mexR* (K44M), *oprD* (nt209Δ1), *mexZ* (V48A), *dacB* (P70L), *gyrA* (T83I), ***ampC* (G183D)**, *ftsI* (G216S), *pmrB* (V344M), *parC* (S87L)
HFIM_1_3	CZA 2 h infusion: 168h	256	64	32	32	16	16	32	32	1	2	OXA-2, *aacA7*, *aacA31*, *aadA6*, *mexR* (K44M), *oprD* (nt209Δ1), *mexZ* (V48A), *dacB* (P70L), *gyrA* (T83I), ***ampC* (G183D)**, *ftsI* (G216S), *pmrB* (V344M), *parC* (S87L)
HFIM_2_1	CZA IC: 120h	256	64	32	32	16	16	32	32	1	2	OXA-2, *aacA7*, *aacA31*, *aadA6*, *mexR* (K44M), *oprD* (nt209Δ1), *mexZ* (V48A), *dacB* (P70L), *gyrA* (T83I), ***ampC* (G183D)**, *ftsI* (G216S), *pmrB* (V344M), *parC* (S87L)
HFIM_2_2	CZA IC: 144h	256	64	32	32	16	16	32	32	0.5	1	OXA-2, *aacA7*, *aacA31*, *aadA6*, *mexR* (K44M), *oprD* (nt209Δ1), *mexZ* (V48A), *dacB* (P70L), *gyrA* (T83I), ***ampC* (G183D)**, *ftsI* (G216S), *pmrB* (V344M), *parC* (S87L)
HFIM_2_3	CZA IC: 168h	256	64	32	32	16	16	16	32	1	2	OXA-2, *aacA7*, *aacA31*, *aadA6*, *mexR* (K44M), *oprD* (nt209Δ1), *mexZ* (V48A), *dacB* (P70L), *gyrA* (T83I), ***ampC* (G183D)**, *ftsI* (G216S), *pmrB* (V344M), *parC* (S87L)

^a^HFIM, hollow-fibre infection model mutants.

^b^P/T4, piperacillin-tazobactam; TAZ, ceftazidime; C/T, ceftolozane-tazobactam; CZA, ceftazidime-avibactam; FEP, cefepime; IMI, imipenem; MER, meropenem; AZT, aztreonam. FDC, cefiderocol; IMR, imipenem-relebactam. Ciprofloxacin, amikacin, tobramycin and colistin were also tested. All the strains resulted resistant for ciprofloxacin, amikacin and tobramycin, and susceptible for colistin.

^c^Acquired mutations are highlighted in bold.

### HFIM

**Figure 1.**
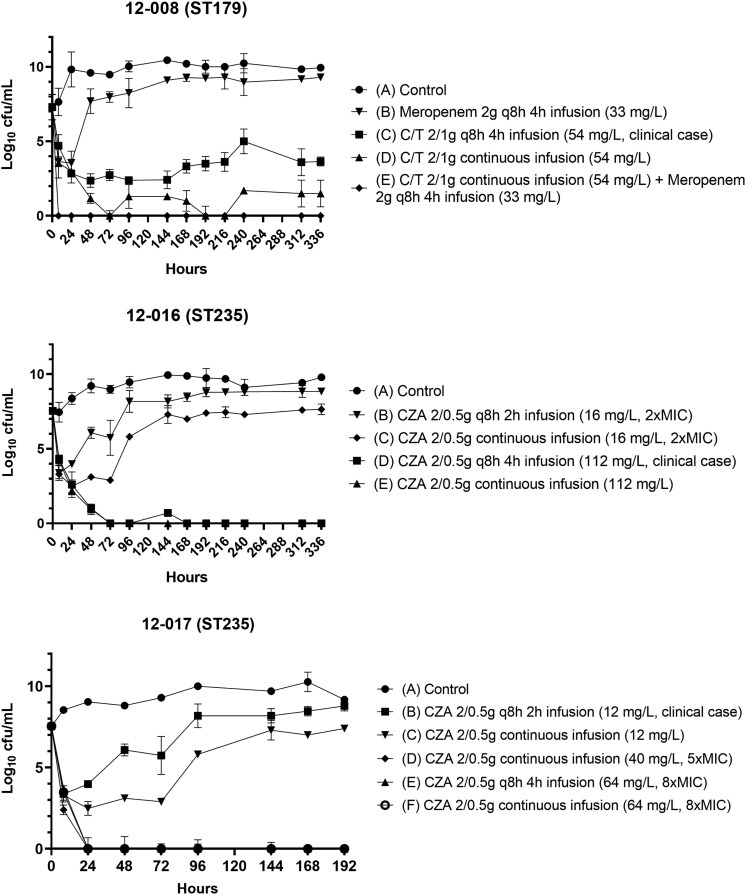
Bacterial density changes (log_10_ cfu/mL) over time for each isolate and treatment in the HFIM. C/T, ceftolozane/tazobactam; CZA, ceftazidime/avibactam; q8h, every 8 h; ST, sequence type.

Bacterial load dynamics were evaluated for isolates 12-008 (ST179), 12-016 (ST235) and 12-017 (ST235) over 8–14 days under different therapeutic regimens (Figure 1). In untreated controls, bacterial density steadily increased, reaching 9.95, 9.80 and 9.18 log_10_ cfu/mL, respectively.

For isolate 12-008 (ST179), ceftolozane/tazobactam administered as a 4-h infusion (2/1 g every 8 h) reduced bacterial load to 2.37 log_10_ cfu/mL at 48 h, but levels stabilized at 3.65 log_10_ cfu/mL by day 14. Continuous infusion (CI) of ceftolozane/tazobactam achieved undetectable bacterial counts by 72 h, although regrowth to 1.5 log_10_ cfu/mL was observed by the end of the experiment. Meropenem (2 g every 8 h, 4-h infusion) induced a transient decrease to 3.54 log_10_ cfu/mL at 24 h, followed by rebound to levels comparable to the untreated control. Combination therapy with ceftolozane/tazobactam CI and meropenem achieved bacterial eradication within 8 h, maintained throughout the experiment.

For 12-016 (ST235) isolate, high-exposure regimens of ceftazidime/avibactam were highly effective. The 4-h infusion (2/0.5 g every 8 h, *C*_max_ 112 mg/L) achieved bacterial eradication by 72 h, although a transient rebound to 0.7 log_10_ cfu/mL was observed at 144 h, followed by complete suppression. CI at the same concentration (*C*_ss_ 112 mg/L), achieved similar results, with bacterial counts reduced to 0.95 log_10_ cfu/mL by 48 h and remaining undetectable thereafter. In contrast, lower-exposure regimens were insufficient to prevent regrowth. A 2-h infusion at 2× MIC (*C*_max_ 16 mg/L) initially reduced the bacterial burden to 3.38 log_10_ cfu/mL within 8 h, but bacterial regrowth occurred reaching 8.85 log_10_ cfu/mL by day 14. CI at the same lower concentration (*C*_ss_ 16 mg/L) showed a comparable trend, with final counts of 8.91 log_10_ cfu/mL.

For isolate 12-017 (ST235), ceftazidime/avibactam (2/0.5 g every 8 h) as a 2-h infusion (*C*_max_ 12) reduced bacterial load to 3.38 log_10_ cfu/mL at 8 h. However, this effect was not sustained, with regrowth reaching 8.79 log_10_ cfu/mL by the end of the experiment. A 4-h infusion at higher exposure (*C*_max_ 64 mg/L; MIC×8) achieved complete eradication within 24 h, with no subsequent regrowth. CI regimens showed exposure-dependent efficacy. CI at *C*_ss_ 12 reduced bacterial load to 2.48 log_10_ cfu/mL at 24 h, followed by regrowth to 7.40 log_10_ cfu/mL. In contrast, higher CI exposures at *C*_ss_ 40 (MIC×5) and *C*_ss_ 64 (MIC×8) achieved bacterial eradication by 24 h and maintained undetectable levels throughout the experiment.

### Resistance studies

Resistance selection was evaluated in the HFIM for isolates 12-008 (ST179), 12-016 (ST235) and 12-017 (ST235) under various therapeutic regimens. The evolution of total and resistant bacterial populations (cfu/mL) over time for each isolate and treatment is presented in the Supplementary Figure, where resistant colony counts are plotted together with total bacterial growth. Changes in resistant subpopulations were analysed by calculating the proportion of colonies growing on antibiotic-supplemented plates (2×–16× MIC) relative to total CFU at each time point.

For 12-008 (ST179), resistant subpopulations emerged under ceftolozane/tazobactam administered as a 4-h infusion (*C*_max_ 54 mg/L), simulating the clinical scenario. Resistance also appeared with meropenem as a 4-h infusion (*C*_max_ 33 mg/L). In contrast, no resistance selection occurred with CI of ceftolozane/tazobactam (*C*_ss_ 54 mg/L), or with the combination of ceftolozane/tazobactam in CI and meropenem.

For 12-016 (ST235), resistant subpopulations emerged with ceftazidime/avibactam administered as a 2-h infusion at 2× MIC (*C*_max_ 16 mg/L), and with CI at the same concentration (*C*_ss_ 16 mg/L). However, no resistant subpopulations were detected with ceftazidime/avibactam administered as a 4-h infusion at high concentrations (*C*_max_ 112 mg/L, clinical case scenario), or as CI at high concentrations (*C*_ss_ 112 mg/L).

For 12-017 (ST235), resistance emerged under low-concentration regimens: ceftazidime/avibactam as a 2-h infusion (*C*_max_ 12 mg/L, clinical case scenario) or as CI (*C*_ss_ 12 mg/L). No resistance selection occurred under high exposures: 4-h infusion at 8× MIC (*C*_max_ 64 mg/L), CI at 5× MIC (*C*_ss_ 40 mg/L) or CI at 8× MIC (*C*_ss_ 64 mg/L).

### Characterization of resistant mutants obtained in the HFIM

The susceptibility profiles and resistance mechanisms detected in the mutants obtained in the HFIM are shown in Tables 2–4, compared to the susceptible-resistant pairs of clinical isolates. As shown, there was an overall high concordance between the mutants obtained *in vivo* and those recovered in the HFIM. Specifically, strain 12-008 (ST179) developed ceftolozane/tazobactam (and meropenem) resistance through the hyperproduction of OXA-10 in both scenarios (Table 2). Likewise, 12-016 (ST235) (Table 3) strain developed ceftazidime/avibactam resistance through the acquisition of extended spectrum mutations in OXA-2 and/or OXA-10. On the other hand, 12-017 (ST235) strain developed resistance in the HFIM through the acquisition of a previously described mutation in the Ω-loop of AmpC (G183D). The activity of cefiderocol and imipenem/relebactam, as potential rescue therapies in cases of ceftolozane/tazobactam and/or ceftazidime/avibactam resistance development, was also assessed. As shown in Tables 2–4, all mutants remained cefiderocol susceptible, even if a significant MIC increase was noted in some of them, particularly for the OXA-10 mutants. Conversely, despite MICs of imipenem/relebactam remained within one 2-fold dilution compared to the parent clinical strain in all mutants, MIC (of both parent and mutants) were frequently located on the edge of the resistance breakpoint (2–4 mg/L).

### Pharmacokinetic validation studies

**Figure 2.**
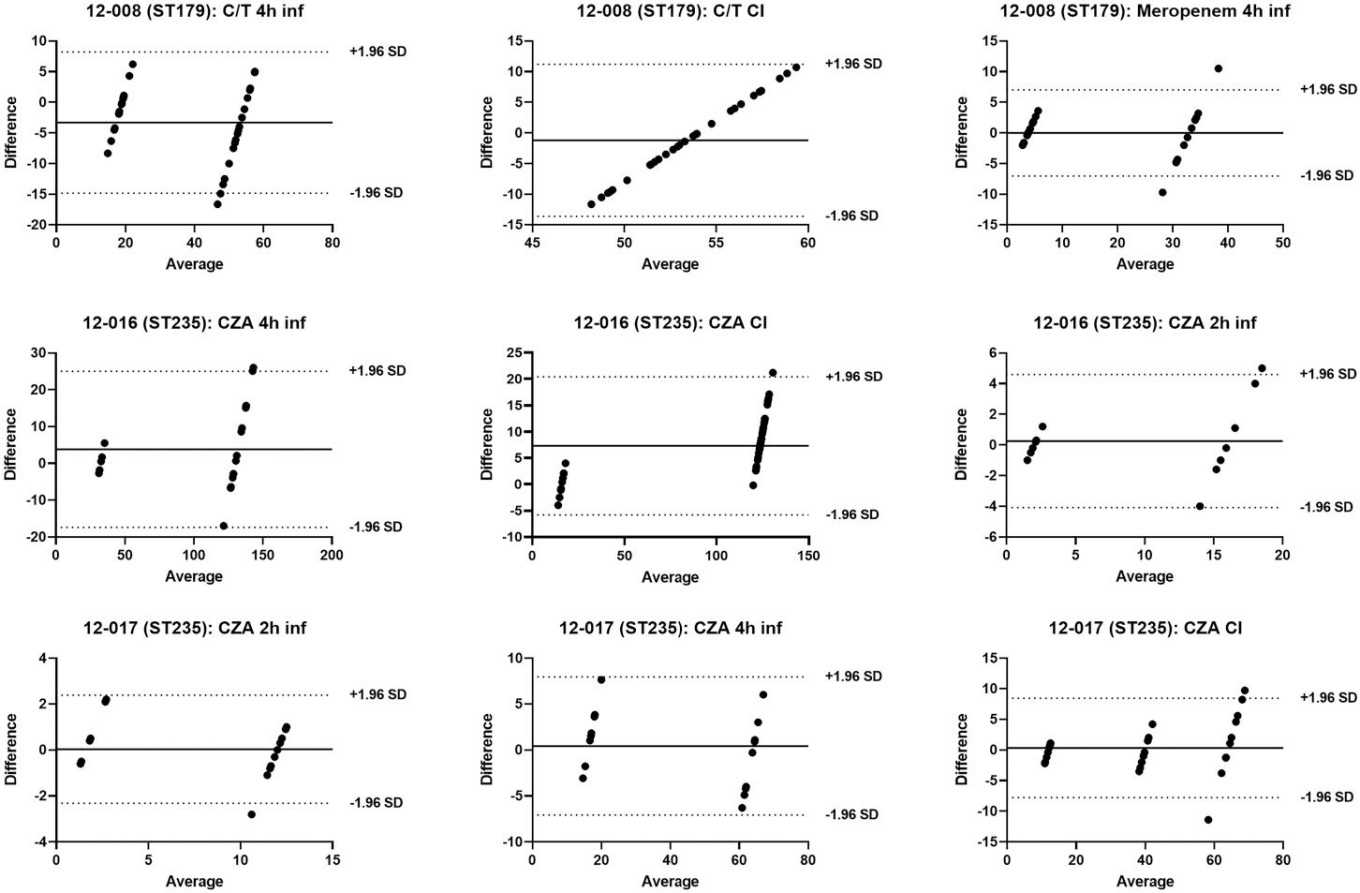
Bland–Altman plot comparing observed and predicted antibiotic concentrations for each regimen across the three isolates. CI, continuous infusion; C/T, ceftolozane/tazobactam; CZA, ceftazidime/avibactam; inf, infusion; SD, standard deviations; ST, sequence type.

The agreement between observed and theoretical antibiotic concentrations was assessed using Bland–Altman plots. Overall, drug exposures were satisfactory, with observed values and standard deviations (SD) closely matching theoretical predictions (Figure 2). For isolate 12-008 (ST179), most values fell within the limits of agreement (±1.96), except for two measurements under the ceftolozane/tazobactam 4-h and meropenem 4-h infusion regimens, which slightly exceeded the range. For isolate 12-016 (ST235), all values were within the limits. Similarly, for isolate 12-017 (ST235), all but one value each from the ceftazidime/avibactam 2-h infusion and CI conditions remained within the ±1.96 SD interval.

## Discussion

XDR *P. aeruginosa* infections represent a therapeutic challenge, particularly in critically ill patients with limited treatment options. Although observational studies suggest that ceftolozane/tazobactam and ceftazidime/avibactam have improved outcomes in this setting, resistance emergence during therapy has been increasingly reported.^12^ Understanding how resistance develops under real-life pharmacokinetic conditions is essential to guide optimal therapeutic strategies and preserve the efficacy of these last-line agents.

In this study, we combined clinical data from three patients with experimental data generated using a HFIM to explore the dynamics and mechanisms of resistance emergence during ceftolozane/tazobactam and ceftazidime/avibactam treatment in OXA-producing *P. aeruginosa*. Our results show a clear association between suboptimal drug exposures and the selection of resistant subpopulations, while optimized dosing regimens prevented both regrowth and resistance development.

Resistance consistently emerged under regimens with low concentrations or short infusion durations. These suboptimal PK/PD conditions allowed bacterial regrowth and promoted the selection of resistant variants, as evidenced by the appearance of colonies on plates supplemented with high concentrations (≥4× MIC) and subsequent MIC increases. In contrast, prolonged or continuous infusion regimens, simulating higher and sustained antibiotic exposures, achieved complete bacterial eradication and prevented the emergence of resistant clones throughout the experiment. These findings align with previous *in vitro* studies that demonstrated improved efficacy and resistance suppression under enhanced exposure conditions.^24^

Recent clinical evidence has highlighted the role of OXA-2 and OXA-10 ESBL variants in mediating resistance to both ceftolozane/tazobactam and ceftazidime/avibactam during treatment. The first report was the acquisition of antibiotic resistance through the selection, during ceftazidime treatment, of an OXA-2 mutation (duplication of amino acid in the Ω-loop leading to OXA-539) in an XDR *P. aeruginosa* infection.^25^ Other reports by Fraile-Ribot^10^ and Arca-Suárez *et al.*^14^ later described the *in vivo* selection of extended-spectrum OXA-10 derivatives in ST235 *P. aeruginosa* isolates from ICU patients. In this work, we confirmed the relevance of the selection of mutations in the Ω-loop region of OXA-2 (W159R, OXA-226) and OXA-10 (G157D, OXA-14 and N143S + G157D, OXA-11) enzymes as a relevant mechanism of resistance development to ceftolozane/tazobactam and ceftazidime/avibactam *in vitro* and *in vivo*. Additionally, for ST179 OXA-10-producing isolates, resistance development, both *in vitro* and *in vivo*, was caused by the overexpression of the β-lactamase instead of the mutation of its Ω-loop. It is noteworthy that this mechanism was not detected through WGS, since as shown in Table 2, the sequences of the susceptible and resistant strains were identical, requiring the elucidation of the involved mechanisms further analysis of gene expression. These findings highlight the challenges for the prediction of susceptibility profiles from WGS resistome analysis.^26^ Indeed, the increase in the copy numbers of the resistance gene or of the genetic element harbouring it has been described as a mechanism for overexpression that should not be neglected.^27^ It is noteworthy that *bla*_OXA_ genes are typically located in transferable genetic elements, including integrons, transposons and/or plasmids underscoring the clinical relevance and potential for horizontal spread.^13^ It is also remarkable that in one case, resistance development to ceftolozane/tazobactam was caused by the selection of an AmpC Ω-loop mutation, stressing the point that OXA-producing strains exhibit a dual potential for resistance development (either AmpC or OXA Ω-loop mutations) as previously noted.^10^ These findings reinforce the need for optimized antibiotic exposure and molecular surveillance and diagnostics to prevent the selection and dissemination of resistant clones.^14^

All PK/PD simulations were performed using the clinical formulations Zerbaxa^®^ (ceftolozane/tazobactam) and Zavicefta^®^ (ceftazidime/avibactam), reproducing real patient treatment conditions. Although the β-lactam and β-lactamase inhibitor components have different pharmacokinetics, exposures were normalized by reproducing the human elimination profile of each compound. The β-lactam (ceftolozane or ceftazidime) was considered the pharmacodynamic driver (%fT > MIC), while the inhibitor (tazobactam or avibactam) was infused at its clinical ratio to ensure β-lactamase inhibition.

This study has several limitations. The number of clinical isolates evaluated was small, and the focus was limited to strains carrying OXA-2 and/or OXA-10 enzymes. Moreover, while HFIM accurately simulates human pharmacokinetics, it does not account for host immune responses or tissue-specific pharmacodynamics, which could influence resistance development *in vivo*. Additionally, antibiotic exposure in the epithelial lining fluid (ELF) compartment has been reported to be significantly lower than in plasma, with ELF/plasma ratios of approximately 30% for ceftazidime and avibactam.^28^ Likewise, the influx and elimination rate constants in ELF were found to be markedly reduced—by approximately 97% for ceftolozane and 52% for tazobactam—in patients with pneumonia.^29^ As the HFIM reproduces plasma pharmacokinetic profiles rather than ELF concentrations, this discrepancy could imply that actual pulmonary exposures are lower than those simulated *in vitro*. Nevertheless, because dosing recommendations and PK/PD targets are based on plasma exposures, our findings remain clinically meaningful for guiding optimized treatment regimens aimed at preventing resistance.

In conclusion, this study shows that resistance to ceftolozane/tazobactam and ceftazidime/avibactam in OXA-producing *P. aeruginosa* can rapidly emerge under suboptimal antibiotic exposure, both clinically and *in vitro*. Optimized dosing strategies, particularly high concentrations and prolonged or continuous infusion, suppressed resistance and achieved bacterial eradication. These findings underscore the importance of individualized, PK/PD-guided therapy and the need to monitor emerging resistance mechanisms, such as extended-spectrum OXA variants, during treatment.

## Supplementary Material

dkaf464_Supplementary_Data

## Transparency declarations

M.M.M. has received consulting fees and participated in educational activities from Pfizer, MSD, Shionogi and Biomerieux. J.P.H. has acted as a consultant to Advanz, Mundipharma, Alifax, Menarini and TFT Pharmaceuticals. He has received honoraria for presentations from Alifax, GSK, MSD and Pfzer, and support for attending meetings from MSD, Pfzer and Shionogi. He has been paid for his attendance at advisory boards and involved in a clinical trial sponsored by DSMB and TFT Pharmaceuticals. A.O. has received fees as a speaker and/or consultant from MSD, Shionogi, Pfizer and Advance; and he also received research grants from MSD and Shionogi. All other authors have no potential conflict of interest.

## Supplementary data

Supplementary Figure and Tables S1 and S2 are available as Supplementary data at *JAC* Online.
