# Supplementary material for: Preventing resistance development in infections by OXA β-lactamase-producing Pseudomonas aeruginosa: correlating clinical outcomes with hollow-fibre model input
Source: J Antimicrob Chemother. 2025 Dec 16;81(1):dkaf464. doi: 10.1093/jac/dkaf464 (PMC12802920; doi:10.1093/jac/dkaf464)
Supplement: dkaf464_Supplementary_Data [file dkaf464_supplementary_data.zip › Supplementary_Figure.docx]

**Supplementary Figure.** Resistant and total colony counts over time for each isolate and treatment in the hollow-fiber infection model.

- **12-008 (ST179)**


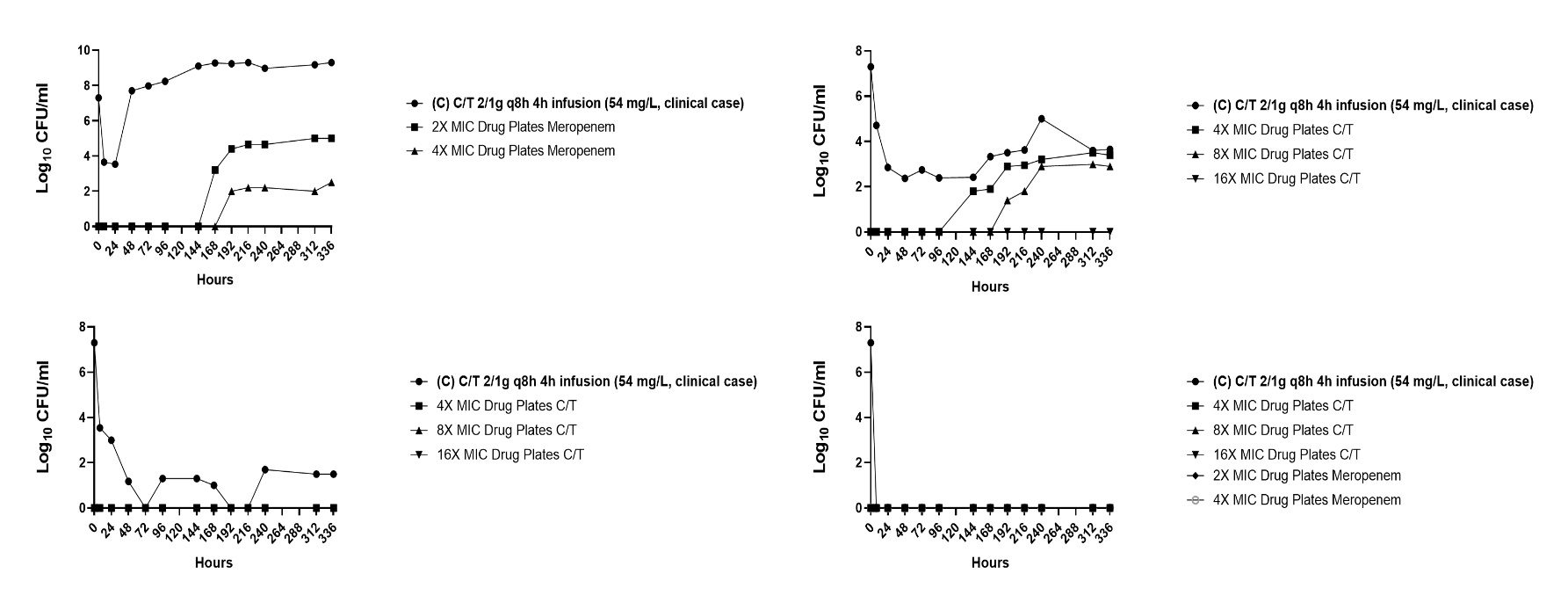


- **12-016 (ST2335)**


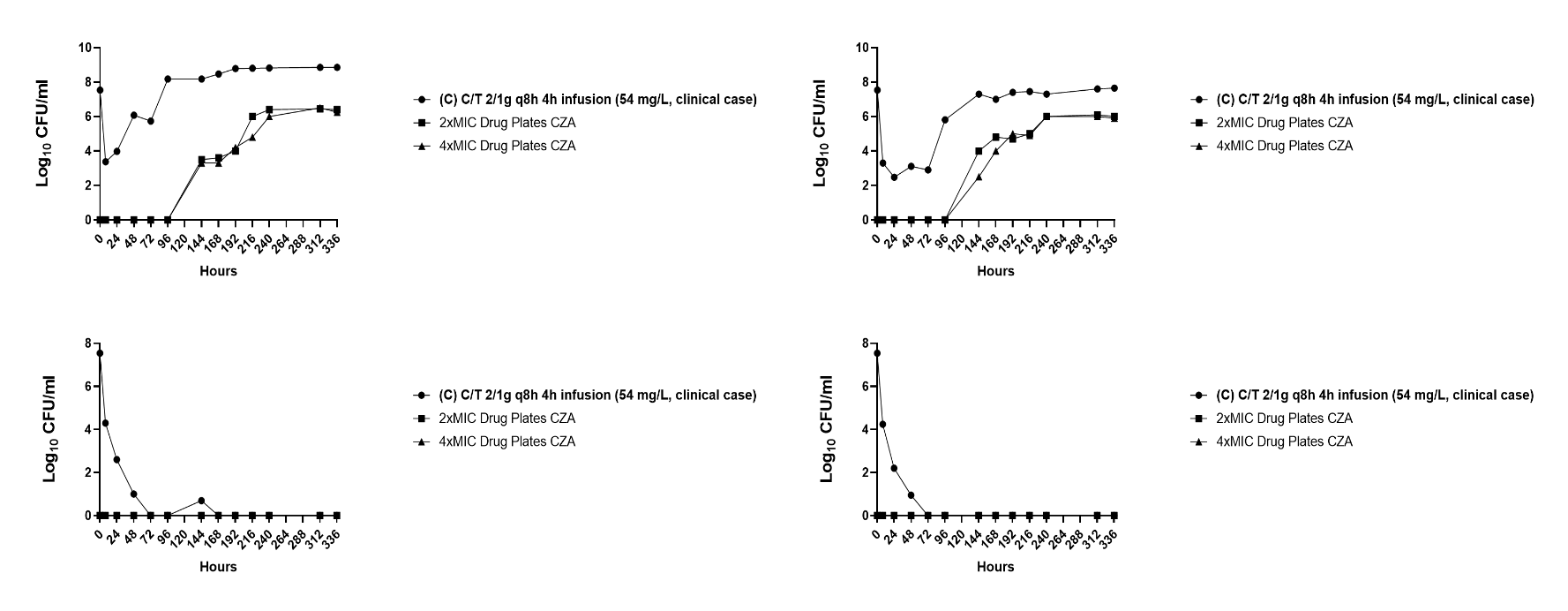


- **12-017 (ST235)**


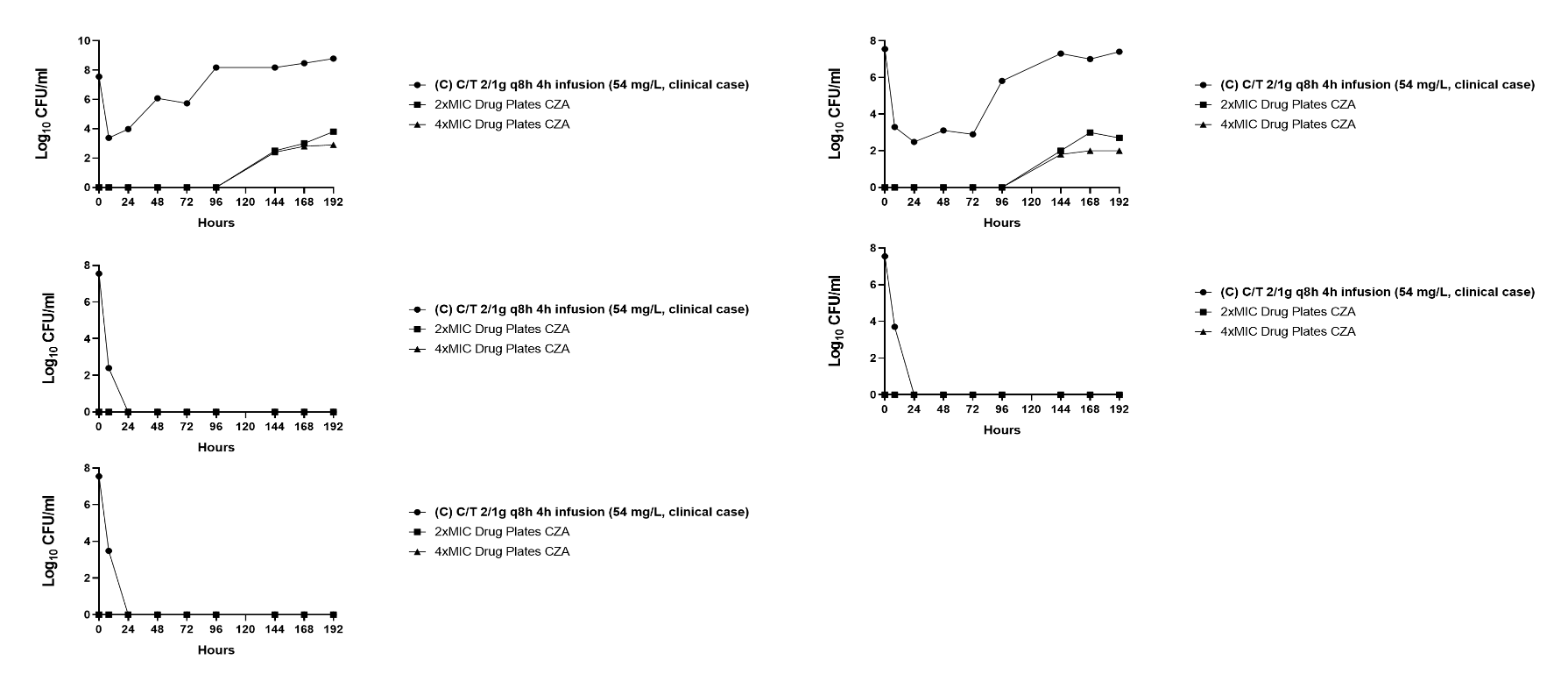


Resistant and total colony counts (log₁₀ cfu/mL) over time for isolates **12-008 (ST179), 12-016 (ST235),** and **12-017 (ST235)** under the different dosing regimens simulated in the hollow-fiber infection model. Resistant colonies were enumerated on TSA plates containing the corresponding antibiotic at 2×, 4×, 8× and 16× the MIC of the parental strain. “Clinical case” indicates the dosing regimen simulated in the HFIM to reproduce the treatment administered to the patient. Abbreviations: C/T, ceftolozane/tazobactam; CZA, ceftazidime/avibactam; CI, continuous infusion; Css, steady-state concentration; MIC, minimum inhibitory concentration.
