# Supplementary material for: Preventing resistance development in infections by OXA β-lactamase-producing Pseudomonas aeruginosa: correlating clinical outcomes with hollow-fibre model input
Source: J Antimicrob Chemother. 2025 Dec 16;81(1):dkaf464. doi: 10.1093/jac/dkaf464 (PMC12802920; doi:10.1093/jac/dkaf464)
Supplement: dkaf464_Supplementary_Data [file dkaf464_supplementary_data.zip › Supplementary_Tables.docx]

**Supplementary Table S1.** Pharmacokinetic sampling schedule used for HFIM validation by regimen.

| Regimen | Sampling times (h)* | Purpose |
| --- | --- | --- |
| Intermittent q8h (2-h infusion) | 0 (Cmin), 2 (EoI/Cmax), 3, 6, 8 (Cmin), 24, 48, 72, 96, 144, 168, 192, 216, 240, 288, 312, 336 | Cmax, Cmin, mid-interval concentrations |
| Intermittent q8h (4-h infusion) | 0 (Cmin), 4 (EoI/Cmax), 5, 8 (Cmin), 24, 48, 72, 96, 144, 168, 192, 216, 240, 288, 312, 336 | Cmax, Cmin, mid-interval concentrations |
| Continuous infusion (CI) | 0, 4, 8, 12, 24, 48, 72, 96, 144, 168, 192, 216, 240, 288, 312, 336 | Css confirmation |

Abbreviations**:** EoI, end of infusion; Cmax, maximum concentration; Cmin, minimum (trough) concentration; Css, steady-state concentration; CI, continuous infusion; q8h, every 8 hours.
The sampling schedule reflects the approach used across experimental branches. Individual HFIM runs included the time points required by the specific regimen and experiment duration (typically 8–14 days, with some runs extended to 312–336 h as indicated in the main text).

**Supplementary Table S2.** Comparison of clinical plasma concentrations and HFIM-simulated concentrations for ceftolozane/tazobactam and ceftazidime/avibactam in the Pseudonova study.

| Patient (Isolate/ST) | Antibiotic | | Dosing regimen | Parameter | Clinical value (mg/L)* | Simulated HFIM value (mg/L) | Difference (%) |
| --- | --- | --- | --- | --- | --- | --- | --- |
| 12-008 (ST179) | | C/T | 2/1 g q8h (4-h infusion) | Cmax | 54 | 50 | 7.4 |
| 12-016 (ST235) | | CZA | 2/0.5 g q8h (4-h infusion) | Cmax | 112 | 123 | 9.82 |
| 12-017 (ST235) | | CZA | 2/0.5 g q8h (2-h infusion) | Cmax | 12 | 11.7 | 2.5 |

Abbreviations**:** C/T, ceftolozane/tazobactam; CZA, ceftazidime/avibactam; Cmax, maximum concentration; q8h, every 8 hours.
