# Supplementary material for: Preventing resistance development in infections by OXA β-lactamase-producing Pseudomonas aeruginosa: correlating clinical outcomes with hollow-fibre model input
Source: J Antimicrob Chemother. 2025 Dec 16;81(1):dkaf464. doi: 10.1093/jac/dkaf464 (PMC12802920; doi:10.1093/jac/dkaf464)
Supplement: Supplementary file 4 [file dkaf464_supplementary_data.zip › Supplementary_Methods.odt]

Online Supplement to: “Preventing resistance development in infections by OXA β-lactamase-producing Pseudomonas aeruginosa: correlating clinical outcomes with hollow-fiber model input”

María M Montero*#1,2,3and Almudena Fernández-Muñoz#4, Sandra Domene-Ochoa1,3, Silvia Castañeda1, Inmaculada López- Montesinos1,3, Sara Cortes-Lara4, Asunción Colomar3,7, Xavier Mulet3,4, Carla López-Causapé3,4, Sonia Luque3,5, Luisa Sorlí1,2,3, Eduardo Padilla2,6, Juan P Horcajada†1,2,3, Antonio Oliver*†3, 4

Supplementary Methods: Characterization of resistance mechanisms

- Whole genome sequencing. Previously defined and validated protocols were used with slight modifications [20]. Total DNA was isolated using a commercial DNA extraction Kit (High Pure PCR Template Preparation Kit, Roche Diagnostics), indexed paired-end libraries were generated using the Illumina DNA Prep library preparation kit (Illumina Inc., USA) and then sequenced on an Illumina NovaSeq 6000® platform. The raw reads for each isolate were mapped against the genome of the P. aeruginosa reference strain PAO1 (RefSeq accession number NC_002516.2) using Bowtie 2 software, version 2.2.6 ([http://bowtie-bio.sourceforge.net/bowtie2/index.shtml](http://bowtie-bio.sourceforge.net/bowtie2/index.shtml)). Pileups and raw files of the mapped reads were obtained using SAMtools, version 0.1.16 ([https://sourceforge.net/projects/samtools/files/samtools/](https://sourceforge.net/projects/samtools/files/samtools/)) and Picard, version 1.140 ([https://github.com/broadinstitute/picard](https://github.com/broadinstitute/picard)). Read alignments surrounding all putative indels were realigned using the Genome Analysis Toolkit (GATK), version 3.4-46 ([https://www.broadinstitute.org/gatk/](https://www.broadinstitute.org/gatk/)). The list of SNPs was compiled from the raw files that met the following criteria: a quality score of >50, a root mean square (RMS) mapping quality of >25 and a coverage depth of >30. Microindels were extracted from the totalpileup files by using the following criteria: a quality score of >250, an RMS mapping quality of >25 and a coverage depth of >30. SNPs and indels for each isolate were annotated by using SnpEff software version 4.3 ([http://snpeff.sourceforge.net/index.html](http://snpeff.sourceforge.net/index.html)). Finally, large chromosomal deletions were analyzed with SeqMonk version 1.47.2 ([https://www.bioinformatics.babraham.ac.uk/projects/seqmonk/](https://www.bioinformatics.babraham.ac.uk/projects/seqmonk/)). Acquired mutations in OXA-2 or OXA-10 were detected mapping raw reads against reference OXA sequences from the ResFinder database and performing an in-depth analysis of the pileup files.

- Quantification of the expression of blaOXA-10. mRNA of blaOXA-10 gene was quantified through real-time RT-PCR according to previously described protocols [21,22] using primers OXA10RNAF (ACCAATGACTTAGCTCGTGCA) and OXA10RNAR (CTTACTTCGCCAACTTCTCTG). Total RNA was extracted with the RNeasy minikit (Qiagen) and treated with 2 U of Turbo DNase (ThermoFisher) for 30 min at 37°C to remove contaminating DNA. Five nanograms of purified RNA was used for one-step reverse transcription and real-time PCR using the QuantiTect SYBR green RT-PCR kit (Qiagen) in a CFX Connect device (Bio-Rad). The rpsL housekeeping gene was used to normalize mRNA levels using previously described primers [22], and the results normalized to parental clinical strain. All RT-PCRs were performed in duplicate, and the mean expression values from three independent RNA extractions were considered.
